# Assessing Prognostic and Predictive Biomarkers of Regorafenib Response in Patients with Advanced Soft Tissue Sarcoma: REGOSARC Study

**DOI:** 10.3390/cancers12123746

**Published:** 2020-12-12

**Authors:** Thomas Brodowicz, Bernadette Liegl-Atzwanger, Nicolas Penel, Olivier Mir, Jean-Yves Blay, Karl Kashofer, Axel Le Cesne, Emilie Decoupigny, Jennifer Wallet, Rainer Hamacher, Marie-Cecile Le Deley

**Affiliations:** 1 Department of Medicine I, Clinical Division of Oncology, Medical University of Vienna–General Hospital, 1090 Vienna, Austria; thomas.brodowicz@meduniwien.ac.at; 2 Diagnostic and Research Institute of Pathology, Comprehensive Cancer Center, Subunit Sarcoma, Medical University of Graz, 8036 Graz, Austria; Bernadette.liegl-atzwanger@medunigraz.at (B.L.-A.); karl.kashofer@medunigraz.at (K.K.); 3 Medical School & Medical Oncology Department, Université de Lille & Centre Oscar Lambret, 59000 Lille, France; 4 Direction of Research and Innovation, Centre Oscar Lambret, 59000 Lille, France; e-decoupigny@o-lambret.fr (E.D.); j-wallet@o-lambret.fr (J.W.); m-ledeley@o-lambret.fr (M.-C.L.D.); 5 Department of Cancer Medicine, Gustave Roussy Cancer Campus, 94800 Villejuif, France; olivier.mir@gustaveroussy.fr (O.M.); axel.lecesne@gustaveroussy.fr (A.L.C.); 6 Department of Medical Oncology, Centre Léon Bérard & Université Claude Bernard Lyon I, 69000 Lyon, France; jean-yves.blay@lyon.unicancer.fr; 7 Department of Medical Oncology, Sarcoma Center, West German Cancer Center, University Hospital Essen, University of Duisburg-Essen, 47057 Duisburg, Germany; rainer.hamacher@uk-essen.de; 8 Paris-Saclay University, Paris-Sud University, UVSQ, CESP, INSERM, 94800 Villejuif, France

**Keywords:** regorafenib, biomarkers, sarcoma, soft tissue sarcoma, prognosis, multikinase inhibitor

## Abstract

**Simple Summary:**

In a double-blind, placebo-controlled, randomized phase 2 trial, Regorafenib provided a clinical benefit to patients with advanced and anthracycline-pretreated soft tissue sarcoma. However, extensive mutational analysis of tumor genes could not identify predictive markers of regorafenib efficacy, including among genes involving in angiogenesis (FLT1, FLT2, FLT3, FLT4, KDR, TEK (TIE2), and VHL). The identification of the precise mechanism of action of multikinase inhibitor in sarcoma and the identification of responding patients requires further clinical studies.

**Abstract:**

Regorafenib significantly prolonged progression-free survival (PFS) in pretreated patients with advanced non-adipocytic sarcoma (HR = 0.46; *p* < 0.001) in a placebo-controlled, randomized, phase-II trial (NCT01900743). Thus, here, we assessed the prevalence of 57 biomarkers and their prognostic and predictive values for PFS and overall survival (OS). We analyzed 134/182 patients included in this trial, treated with regorafenib (*n* = 71, 53%) or placebo (*n* = 63, 47%). Mutational analyses were performed via full coding sequence analysis for 10 genes, and mutation hotspot panel for 50 genes (four genes in common). H19 was studied with RNA in-situ hybridization. The prognostic and predictive biomarkers’ values were studied only for biomarkers found positive/mutated in at least 10 patients. Overall, 25 out of 57 studied biomarkers, including five out of seven genes involved in angiogenesis, were found mutated/positive in at least one patient, of which 23 biomarkers had low prevalence (fewer than eight out of 134 patients), contrasting with H19 (*n* = 24, 18%), and TP53 (*n* = 35, 26%). However, in multivariable models of PFS and OS, including treatment effects and interactions, no significant prognostic or predictive values of the tested biomarkers were observed. Though several promising biomarkers were found to be positive/mutated, none of them were identified as viable predictive and prognostic biomarkers.

## 1. Introduction

Soft tissue sarcomas (STS) are rare heterogeneous malignant tumors of mesenchymal origin, with an estimated incidence averaging 4–5/100,000/year in Europe. STS accounts for approximately 2% of all adult tumors and 4–8% of pediatric malignancies. At least 40% of the patients with STS develop recurrent or metastatic incurable disease. For decades, palliative chemotherapy with anthracycline (usually doxorubicin) either as monotherapy, or in combination with ifosfamide has been the conventional first-line systemic therapy for metastatic disease, which provides an overall survival (OS) of approximately 18 months. However, doxorubicin-induced cumulative cardiotoxicity prevents protracted treatments and re-treatments in most cases [1]. Although several newly-approved second- and further-line drugs such as ifosfamide [2], dacarbazine, trabectedin, pazopanib [3], and eribulin, have shown some signs of activity, there is no consensual treatment after failure, or intolerance of doxorubicin. The prognosis of patients with doxorubicin-refractory metastatic STS remains poor, and there remains a large unmet treatment need for such patients.

Angiogenesis is of crucial importance for tumor growth and dissemination of malignancies, and it mainly depends on vascular endothelial growth factors (VEGF) and other pro-angiogenic factors. Consequently, angiogenesis inhibition has been identified as active strategy for the treatment of metastatic STS. A study with pazopanib, an oral, multi-targeted, tyrosine kinase inhibitor has reported promising results in patients with metastatic non-adipocytic STS after failure of standard chemotherapy. Regorafenib (BAY 73-4506, Stivarga^®^, Bayer, Leverkusen, Germany) is another oral tumor deactivation agent that potently blocks multiple protein kinases, including those involved in tumor angiogenesis (VEGFR receptor 1, −2, −3, TIE2), oncogenesis (KIT, RET, RAF-1, BRAF, BRAFV600E), metastasis (VEGF receptor 3, PDGFR, FGFR), and tumor immunity (CSF1R). In addition, regorafenib reduces the levels of tumor-associated macrophages, which induces changes in the tumor microenvironment, contributing to its antitumor activity [4,5]. Furthermore, the present study is the ideal framework to better explore the prognosis/predictive value of an emergent biomarker in sarcoma: H19 [6].

We previously reported that regorafenib significantly improved progression-free survival (PFS) in patients with non-adipocytic STS in a randomized, double-blind, placebo-controlled, phase II REGOSARC trial (NCT01900743), which assessed the activity and safety of regorafenib in doxorubicin-refractory metastatic STS [7]. This pre-planned translational study had the following objectives: (1) to identify, and characterize the molecular mutations in such patients, (2) to explore the prognostic values of the potential biomarkers and their predictive values for response to regorafenib, and (3) to better define the patients more sensitive to regorafenib.

## 2. Methods

### 2.1. Study Design

The current translational research was embedded within the REGOSARC trial. Full details of REGOSARC trial have been reported earlier [7,8] (see Appendix A).

Within the four cohorts, patients were centrally randomly assigned in 1:1 ratio to receive either oral regorafenib (160 mg/day, 3 weeks on/1-week off) plus best supportive care (BSC) or matched placebo plus BSC until confirmed progressive disease as per RECIST version 1.1, unacceptable toxicity, death, patient’s refusal of treatment, or investigator’s decision. Randomization was stratified by country (France or Austria), and prior exposure to pazopanib (yes or no). Patients receiving placebo who experienced disease progression (centrally confirmed according to RECIST version 1.1) could be offered optional crossover to regorafenib on an open-label basis. During the treatment period, the tumor assessments were performed monthly during the first 4 months, and every 3 months thereafter until intolerance or disease progression. 

All study procedures were conducted in accordance with the International Council for Harmonization tripartite guideline on good clinical practice and were approved by the institutional ethical and regulatory committees of each participating center Number NCT01900743. Signed informed consents were obtained from all study participants before registration.

### 2.2. Translational Research

Central translational analyses were performed by the Diagnostic and Research Institute of Pathology Medical University Graz in Austria with an objective to investigate the mutational status (describe which type of mutations: missenses, translocations, amplifications, do you also have the number off gene copies if <to amplification ) in targeted genes as well as in 50 oncogenes and tumor suppressor genes commonly mutated in cancer with the aim to explore potential prognostic and predictive factors for treatment response. 

To centrally confirm the diagnosis, we reviewed in all patients the chromosomal aberrations characteristic for specific sarcoma subtypes via immunohistochemistry (IHC), molecular analysis (fluorescence in situ hybridization (FISH), and reverse transcriptase polymerase chain reaction (RT-PCR)). For this, collection of FFPE tumor blocks, or fresh frozen tissue samples was mandatory for all patients, either from the primary tumor or from metastatic sites (or both). All tumor samples were obtained prior to any treatment with regorafenib, and accompanied by electronic information on patient characteristics, histopathology reports, and if applicable, the written reports on previously performed molecular analysis of the tumor.

Since regorafenib is a large-spectrum multi-kinase inhibitor, we conducted wide analysis, including kinases involved in angiogenesis, other receptor and non-receptor kinases, second messengers, and genes. A mutational analysis was performed via full coding sequence analysis for the following 10 genes: FLT1 (VEGFR1), FLT2 (FGFR1), FLT4 (VEGFR3), KDR (VEGFR2), KIT, PDGFRB, RAF1, TEK (TIE2), TP53, and VSX2 (RET1). In addition, mutational analysis was performed via a mutation hot spot panel, Ion AmpliSeq™ Cancer Hotspot Panel v2, Thermo Fisher Scientific (CHP2), covering 50 genes: 4 genes in common with the full coding sequence analysis (FLT2, KDR, KIT and TP53), and 46 additional genes (ABL1, AKT1, ALK, APC, ATM, BRAF, CDH1, CDKN2A, CSF1R, CTNNB1, EGFR, ERBB2, ERBB4, EZH2, FBXW7, FGFR2, FGFR3, FLT3, GNA11, GNAQ, GNAS, HNF1A, HRAS, IDH1, IDH2, JAK2, JAK3, KRAS, MET, MLH1, MPL, NOTCH1, NPM1, NRAS, PDGFRA, PIK3CA, PTEN, PTPN11, RB1, RET, SMAD4, SMARCB1, SMO, SRC, STK11, and VHL). For these 56 genes, results were coded as mutated versus wild type. Of the above genes, we considered together the seven genes involved in angiogenesis: FLT1, FLT2, FLT3, FLT4, KDR, TEK (TIE2) and VHL, leading to a new variable noted “Angiogenesis pool” coded “mutated” if at least one of the seven genes of the pool was mutated. Lastly, H19 was studied on the tissue micro-array (TMA) via RNA in situ hybridization. Results of H19 (TMA) were coded as positive versus negative. 

### 2.3. Statistical Analysis

The analysis was based on all patients enrolled in the cohort A, B, C, or D of the REGOSARC trial, provided they had biomarkers’ evaluation (*n* = 134). The main objective of this translational study analysis was to evaluate the prognostic value, and the predictive value of the biomarkers in terms of progression-free survival (PFS, main endpoint), and overall survival (OS, secondary endpoint). Progression-free survival times were calculated from the date of randomization until the date of progression or death, from any cause; patients who were alive free of progression at last follow-up were censored at the time of last visit. Overall survival times were calculated from the date of randomization until the date of death, from any cause; patients who were alive at last follow-up were censored at the time of last visit. 

Kaplan–Meier curves for PFS and OS were estimated according to the biomarker status (positive versus negative for H19, mutated versus wild type gene for the other biomarkers). Prognostic impacts of H19 (positive versus negative), or genes (mutated versus wild type) on PFS (or OS) were evaluated by the hazard ratio of progression (or death) in Cox models adjusted by the treatment effects (Regorafenib versus Placebo). These analyses were performed only for the biomarkers with enough patients with a mutation (more than 10 patients). The predictive value of each evaluated biomarker was assessed by evaluating the heterogeneity of treatment effect according to the biomarker status, in Cox models, including the following factors: (i) treatment arm effect (Regorafenib versus Placebo), (ii) biomarker effect (positive versus negative, or mutated versus wild type) and (iii) interaction between treatment arm and biomarker. These latter results were illustrated with Forest plots. For the different Cox models, the proportional hazards assumptions were evaluated using Schoenfeld residuals. The significance threshold was set at 5% two-sided. Estimates were provided with their 95% confidence intervals (95% CI). Statistical analyses were conducted using STATA, version 13.1 statistical software (StataCorp, LP, College Station, TX, USA). 

## 3. Results

### 3.1. Patient Disposition and Disease Characteristics

This translational research analyzed 134 of the 182 patients enrolled in the REGOSARC trial, since the tissue samples from the other 48 patients were not available for the analyses. As detailed in Table 1, median age was 59 years (range, 21–81) and there were 71 women (53%); 71 patients (53%) were treated with regorafenib and 63 patients (47%) were treated with placebo. Baseline characteristics were well balanced in both arms and comparable to those reported in the overall population 7 (Table 1). 

### 3.2. Biomarker Distribution

Considering the 50 genes evaluated via HotSpot analysis, no mutations were found for 33 genes, whereas a mutation was reported in at least one tumor for the remaining 17 genes. For the genes also evaluated via full sequence, all HotSpot mutations were confirmed via the full sequence analysis (3 genes: KDR, KIT, and TP53). In addition, for each of the 10 genes evaluated by full sequence analysis, a mutation was reported in at least 1 tumor, including FGFR1 that was found mutated in two tumors, although these was not identified via the HotSpot analysis. As detailed in Table 2 and Table S1, most mutations were rare with the mutations being observed in less than eight of 134 tumors for 23 genes: AKT1, ATM, BRAF, CDKN2A, CTNNB1, FBXW7, FLT2, FGFR3, FLT1, FLT4, HRAS, KDR, KIT, MET, NRAS, PDGFRB, PIK3CA, PTEN, RAF1, RB1, SMARCB1, TEK (TIE2), and VSX2 (RET1). 

This contrasted with TP53, mutated in 35 tumors (26%). TP53 mutation was significantly more frequent in leiomyosarcoma (16/45, 36%), and other sarcomas (13/34, 38%) rather than in liposarcoma (5/38, 13%), and synovial sarcoma (1/17, 6%) (*p* = 0.009). At least one of the seven genes considered in the angiogenesis pool was mutated in 20 tumors (15%), with no significant difference between histological subgroups (*p* = 0.42): five tumors with each FLT1 and FLT4 mutations, two tumors with FLT2 mutation, seven tumors with KDR mutation, three tumors with TEK (TIE2) mutation, and no tumors with FLT3 and VHL mutations. This includes two tumors with two mutations: one tumor with both FLT1 and KDR mutations and one tumor with both TEK (TIE2) and KDR (VEGFR2) mutations. Lastly, a positive H19 was observed in 24 tumors (18%). The proportion of H19 positivity significantly differed between histological subgroups, varying from three out of 34 (9%) in sarcomas other than liposarcoma, leiomyosarcoma and synovial sarcoma, to seven of 17 (41%) in synovial sarcoma (*p* = 0.02).

### 3.3. Survival Outcomes

**Figure 1:**
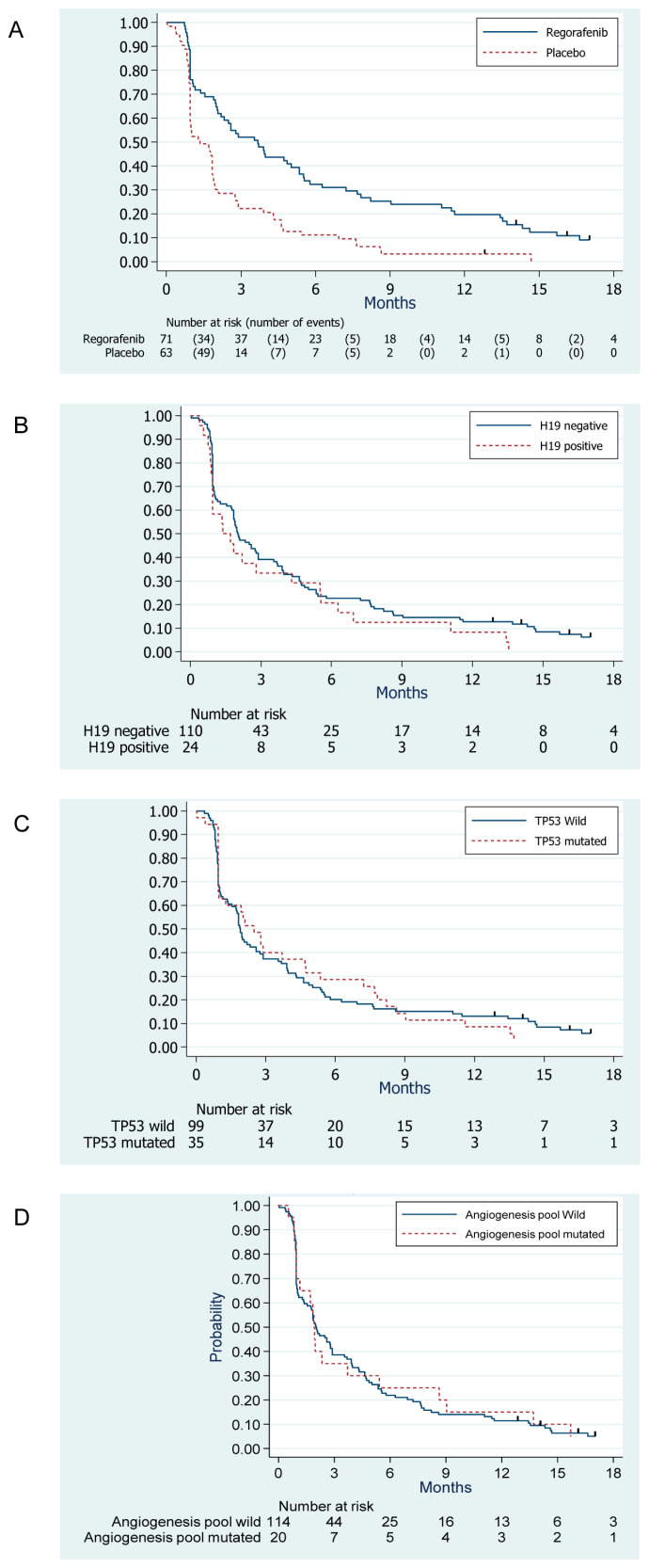
Kaplan-Meier estimates of progression-free survival curves according to treatment arm (**A**), H19 status (**B**), *TP53* mutation (**C**), and presence of mutation in the angiogenesis pool (**D**).

At the time of the analysis, 127 events were reported among the 134 study patients: 126 patients had a disease progression, and 1 died from an unrelated cause. The median PFS was 3.7 months (95% CI, 2.1–5.4) and 1.3 months (95% CI, 1.0–1.8) in the Regorafenib arm and placebo arm, respectively (Figure 1A). 

Compared to placebo, we observed a significant benefit associated with regorafenib in terms of PFS, with a HR = 0.46, 95% CI, 0.32–0.67, *p*-value < 0.001 in the univariate analysis, which was consistent with the results published on the whole randomized population [7]. Similar to the results reported in the whole randomized population, there was no statistically significant difference in the OS between the two arms: the median OS was 12.1 months (95% CI, 7.2–16.6) and 9.0 months (95% CI, 7.5–12.8), in the Regorafenib arm and placebo arm, respectively; HR = 0.85, 95% CI, 0.56–1.29, *p* = 0.45.

### 3.4. Evaluation of the Prognostic Value of Biomarkers

As detailed in Table 3 and illustrated by Figure 1B–D, we did not observe any significant prognostic value of TP53 mutation, H19 positivity, and presence of at least one mutation in the angiogenesis pool in terms of PFS. Similar results were observed with OS (Table 3). 

### 3.5. Evaluation of the Predictive Value of Biomarkers

**Figure 2:**
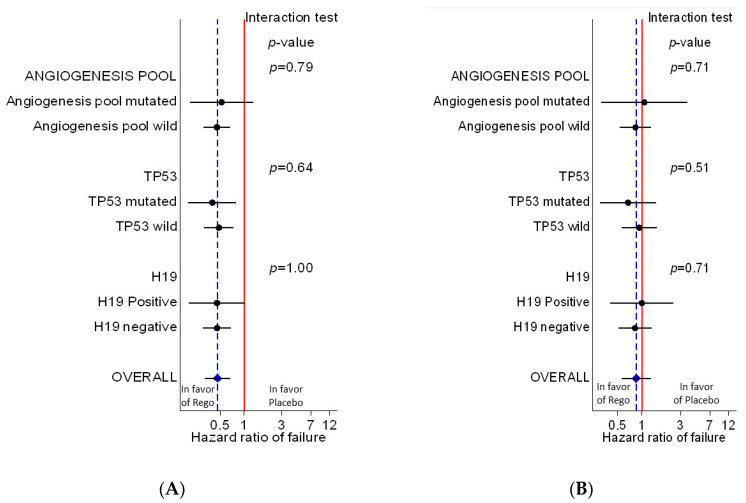
Forest plot (**A**) (for progression-free survival) and (**B**) (for overall survival) showing the heterogeneity of treatment effect according to the status of each biomarker.

We did not observe any significant heterogeneity of treatment effect in terms of both progression free survival (Figure 2A) and overall survival (Figure 2B) according to the status of the three studied biomarkers (Table 3). 

## 4. Discussion

Most tumors are associated with complex genetic alterations such as gains, losses, and point mutations. In this translational study, we hypothesized that inter-individual variability of responses to regorafenib might be predominantly associated with alterations of genes responsible for encoding the protein kinases specifically targeted by regorafenib. Therefore, we conducted an extensive translational research to identify predictive factors. However, we found no significant differences according to the status of explored genes and/or biomarkers in clinical outcomes in the STS patients receiving regorafenib. 

Multikinase inhibitor regorafenib affects multiple enzymes in cancer cells and tumor stroma. Regorafenib in particular blocks multiple membrane-bound and intracellular kinases involved in normal cellular functions, which contribute to neoplastic transformation and progression. Yet, predictive criteria for response to regorafenib remain undetermined. Moreover, validated predictive biomarkers for multi-tyrosine kinase inhibitors efficacy, in general, remain unknown. To date, several approaches to identify biomarkers have met with minimal successes in predicting response to treatment with several multiple tyrosine kinase inhibitors in advanced colorectal cancer and renal cell carcinoma. Therefore, a general paradigm to identify prognostic and predictive markers for response to multikinase inhibitors would be of high clinical value. Once known, those results would have important implications, not only as useful tools to predict prognosis and response to therapy in cancer patients, but also for a better selection of patients, and for the identification of patients that may benefit the most from regorafenib in the routine clinical setting.

In this study, although investigating tumor samples for common hot spot mutations in 50 well established oncogenes and tumor suppressor genes and as well as searching for mutations in the full coding sequence of Regorafinib targets we found no recurrent mutations in a significant subset of cases. Therefore, we evaluated three biomarkers, i.e., H19, TP53, and angiogenesis pool, for their prognostic and predictive abilities because only they were found in a sufficient number of patients (more than 10 patients). In the present study 18% of sarcomas were found positive for the long non-coding RNA H19 by Tissue-Micro-Array. Moreover, the tumors associated with H19 expression seemed to be associated with poor overall survival (HR = 1.60; *p* = 0.07; see Table 3), nevertheless, because of sample size this difference did not reach the classical level of significance. However, H19 expression had no predictive value in our sample. TP53 mutations are one of most common mutations found in soft tissue sarcomas. According to the method used, the frequency of TP53 mutations is around 10–30%. As an example, we found that frequency of TP53 mutations in liposarcomas is about 13%. These mutations occurred in pleomorphic and myxoid/round cell liposarcomas, and not in well differentiated/dedifferentiated liposarcomas. Most of these mutations are likely somatic ones [9], since in the present study, the frequency of TP53 mutation was 26% whereas, in a large study of 559 sarcoma patients, Mitchell et al. found that the frequency of TP53 germline mutations is about 3.6% [10]. The role of TP53 is well known in maintaining genomic integrity. However, this biomarker had no prognostic or predictive value in our sample. We also hypothesized that mutations in key angiogenetic factors could identify patients benefiting from regorafenib. However, mutations in FLT1, FLT4, FLT2, KDR, and TEK (TIE2) were neither prognostic nor predictive. This suggests that the mechanism of action of regorafenib in sarcoma is likely more subtle and complex than we hypothesized. 

Since regorafenib is an active drug in soft tissue sarcoma, bone sarcoma [11], and GIST [12], further clinical explorations are needed to better understand its mechanism of action and identify predictive factors. We have previously reported a clinically meaningful PFS-benefit of regorafenib compared to placebo in anthracycline-pretreated patients with advanced non-adipocytic sarcoma [8]. In addition, a post-hoc exploratory analysis of REGOSARC data provided an integrated measure of regorafenib’s clinical benefit by using the quality-adjusted time without symptoms of progression or toxicity (Q-TWiST) analysis. The results of the Q-TWiST analysis additionally confirmed that regorafenib offers significantly improved quality-adjusted survival and quality of life outcomes in comparison with a placebo. 

The present translational research study had some limitations. Principally, the tumor sample obtained at baseline might not represent the actual genotype after multiple lines of treatment. In addition, although the REGOSARC study population should be considered representative of patients with advanced STS, this analysis was exploratory; subgroups comparisons were restricted due to the relatively small sample size and low frequency of most mutations, and the power of the study was limited. Additionally, heterogeneity of histological subtypes may complicate the interpretation of the findings, and the analysis by histological subtype was not feasible due to the small number of patients in each cohort. Finally, the sample size did not allow analysis of prognostic/predictive value according to the type of mutations for each gene. We have to dichotomize the molecular analysis in two groups: all mutations together versus wild type gene. 

REGOSARC study is an academic trial with limited funding, this trial did not mandatory biopsy at baseline and during regorafenib treated, consequently, the translational research was focused on mutational analysis of biopsy obtained at diagnosis. Since there was no biopsy at study entry, we observed that in 25% of cases, the mutational analysis was not feasible. We failed to identify predictive or prognostic biomarkers. Some could suggest that additional analysis could be done, as an example, regarding the mechanism of action of regorafenib on kinases of both cancer cells and microenvironmemt, a high throughput phosphor-proteomic analysis is appealing but this requires additional funding. This funding could be helpful to apply more innovative approaches, such as single cell sequence analysis, CRISPR/Cas-based phenotypic studies, or a biomarker discovery platform based on culture cells and animal models.

## 5. Conclusions

To conclude, in this patient population, none of examined biomarkers were revealed as viable predictive and prognostic biomarkers for regorafenib clinical benefit. Increased efforts are warranted to identify and validate wide-ranging biomarkers as candidates for regorafenib efficacy in pretreated patients with advanced non-adipocytic sarcoma.

## Figures and Tables

**Table 1:** Patient and disease characteristics at baseline.

Characteristics		Regorafenib *n* = 71		Placebo *n* = 63		Total *n* = 134	
Sex	Male	32	45%	31	49%	63	47%
	Female	39	55%	32	51%	71	53%
Country	France	64	90%	58	92%	122	91%
	Austria	7	10%	5	8%	12	9%
ECOG PS	0	33	46%	29	46%	62	46%
	1	38	54%	33	52%	71	53%
	2	0	0%	1	2%	1	1%
Age	Median (range)	59	(21–81)	59	(22–80)	59	(21–81)
Histology	Liposarcoma (Cohort A)	18	25%	20	32%	38	28%
	Leiomyosarcoma (Cohort B)	23	32%	22	35%	45	34%
	Synovial sarcoma (Cohort C)	10	14%	7	11%	17	13%
	Other sarcomas (Cohort D) *	20	28%	14	22%	34	25%
Tumor grade (FNCLCC)	Grade 1	7	10%	4	6%	11	8%
	Grade 2	18	25%	35	56%	53	40%
	Grade 3	34	48%	17	27%	51	38%
	Not documented	12	17%	7	11%	19	14%
Primary site	Retroperitoneal	24	34%	24	38%	48	36%
	Lower limb	19	27%	18	29%	37	28%
	Uterus	10	14%	7	11%	17	13%
	Trunk	8	11%	3	5%	11	8%
	Viscera	3	4%	6	10%	9	7%
	Upper limb	1	1%	3	5%	4	3%
	Head and neck	4	6%	0	0%	4	3%
	Other	2	3%	2	3%	4	3%
Staging	Metastatic disease	69	97%	61	97%	130	97%
	Metastatic sites **						
	Lung	46	65%	48	76%	94	70%
	Other	30	42%	31	49%	61	46%
	Peritoneal	24	34%	18	29%	42	31%
	Liver	21	30%	20	32%	41	31%
	Lymph nodes	19	27%	12	19%	31	23%
	Bones	11	15%	13	21%	24	18%
	Cutaneous	6	8%	6	10%	12	9%
	Pleural	5	7%	5	8%	10	7%
Prior treatments	Prior surgery	68	96%	59	94%	127	95%
	Prior radiotherapy	35	49%	38	60%	73	54%
	Prior chemotherapy	71	100%	63	100%	134	100%
Number of prior chemotherapy lines (neoadjuvant, adjuvant and advanced)	Median (range)	2	(1–4)	2	(1–9)	2	(1–9)
	1	24	34%	19	30%	43	32%
	2	25	35%	24	38%	49	37%
	3	20	28%	14	22%	34	25%
	4	2	3%	5	8%	7	5%
	9	0	0%	1	2%	1	1%
Prior treatments (≥10% of patients) ***	Doxorubicin	71	100%	60	95%	131	98%
	Ifosfamide	41	58%	37	59%	78	58%
	Trabectedin	30	42%	23	37%	53	40%
	Gemcitabine	9	13%	18	29%	27	20%
	Dacarbazine	6	8%	14	22%	20	15%

* Other sarcomas eligible in cohort D were adult fibrosarcoma, alveolar soft part sarcoma, angiosarcoma, clear cell sarcoma, endometrial stromal tumor, epithelioid hemangioendothelioma, epihelioid sarcoma, extraskeletal myxoid chondrosarcoma, extraskeletal osteosarcoma, fibrosarcoma, hemangiopericytoma, inflammatory myxofibroblastic sarcoma, intimal sarcoma, low grade fibromyxoid sarcoma, malignant glomus tumor, malignant peripheral nerve sheath tumors, malignant mesenchymoma, malignant solitary fibrous tumor, mesenchymal chondrosarcoma (soft tissue), myxofibrosarcoma, pleomorphic rhabdomyosarcoma, rhabdoid tumor, and undifferentiated pleomorphic sarcoma. ** Patients could have more than one metastatic site. *** Other prior treatments included cyclophosphamide (8%), paclitaxel (4%), pazopanib (3%), and cisplatin (2%), palifosfamide (2%) and docetaxel (2%). ECOG: Eastern Cooperative Oncology Group; FNCLCC: Fédération Nationale des Centres de Lutte Contre le Cancer; PS: performance status.

**Table 2:** Frequency of mutation or positivity for H19, *TP53*, and angiogenesis pool.

Gene	Assessment Technique	Overall *N* = 134	Lipo-Sarcoma *N* = 38	Leiomyo-Sarcoma *N* = 45	Synovial Sarcoma *N* = 17	Others *N* = 34	*p*
*TP53*	HotSpot + FCS ^(1)^	35 (26%)	5	16	1	13	0.009
Angiogenesis pool ^(2)^		20 (15%)	7	5	1	7	0.42
H19	RNA in situ hybridization on TMA	24 (18%)	9	5	7	3	0.02

FCS: Full coding sequence analysis TMA: Tissue Micro-Array ^(1)^ For *TP53*, studied by both techniques, 35 tumors were found mutated, 29 via both techniques and 6 only by FCS ^(2)^ The “Angiogenesis pool” includes *FLT1* (*VEGFR1*), *FLT2* (*FGFR1*), *FLT3*, *FLT4* (*VEGFR3*), *KDR* (*VEGFR2*), *TEK* (*TIE2*), and *VHR*.

**Table 3:** Evaluation of the prognostic and predictive values of H19 positivity, *TP53* mutation, and mutation in angiogenesis pool, in terms of PFS and OS.

Outcome	Prognostic Value ^(1)^		Predictive Value ^(2)^		
Gene	HR (Abnormal vs. normal)	*P*	HR (Rego. vs. plac.) if abnormal	HR (Rego. vs. plac.)if normal	*p* interaction test
Progression-free survival					
H19	1.41 (0.90–2.20)	0.14	0.46 (0.20–1.03)	0.46 (0.30–0.68)	1.00
*TP53*	1.08 (0.72–1.60)	0.72	0.40 (0.20–0.80)	0.48 (0.32–0.74)	0.64
Angiogenesis pool	0.96 (0.59–1.57)	0.88	0.52 (0.21–1.31)	0.45 (0.31–0.67)	0.79
Overall survival					
H19	1.60 (0.96–2.68)	0.07	1.00 (0.40–2.53)	0.82 (0.51–1.32)	0.71
*TP53*	1.03 (0.64–1.65)	0.89	0.67 (0.30–1.49)	0.92 (0.56–1.52)	0.51
Angiogenesis pool	0.73 (0.39–1.38)	0.33	0.83 (0.53–1.31)	1.07 (0.31–3.66)	0.71

HR: Hazard ratios, provided with their 95% confidence intervals; Abnormal: positivity (for H19) or mutation (for *TP53* and angiogenesis pool); Normal: negativity (for H19) or wild type (for *TP53* and angiogenesis pool); Rego: regorafenib arm; plac: placebo arm; HR (Abnormal vs. normal) is the hazard ratio of the subset of patients with positivity or mutation compared to the subset of patients with negativity or wild type. HR (Rego. vs. plac) is the hazard ratio of the regorafenib-arm compared to placebo-arm. The “Angiogenesis pool” includes *FLT1* (*VEGFR1*), *FLT2* (*FGFR1*), *FLT3*, *FLT4* (*VEGFR3*), *KDR* (*VEGFR2*), *TEK* (*TIE2*) and *VHR*. ^(1)^ The prognostic value of each biomarker listed in the table on the risk of progression (upper table) or death (lower table) is estimated for each biomarker separately in a Cox model adjusted for the treatment effect. ^(2)^ The predictive value of each biomarker listed in the table is evaluated for each biomarker separately in a Cox model adjusted for the treatment effect, including an interaction term between the biomarker status and the treatment effect.

## Supplementary Materials

The following are available online at https://www.mdpi.com/2072-6694/12/12/3746/s1, Table S1: Frequency of mutation or positivity for the 57 studied biomarkers.

## Appendix A

REGOSARC was a double-blind, placebo-controlled, multicenter, randomized phase II trial assessing the activity, and safety of regorafenib in patients with four main histological subgroups of STS: liposarcoma (cohort A), leiomyosarcoma (cohort B), synovial sarcoma (cohort C), and other sarcomas (cohort D). Eligible patients were adults (≥18 years old) with metastatic and/or unresectable STS, measurable lesions in accordance with RECIST version 1.1, documentation of progression within the last six months before the enrolment, and reported failure or intolerance to doxorubicin or other anthracycline-based chemotherapy (up to three prior lines). Particularly, the central confirmation of histopathological diagnoses of STS with available formalin fixed paraffin embedded (FFPE) blocks was mandatory for all patients at baseline (if not previously performed in reference centers). Other eligibility criteria included an ECOG performance status of 0 to 1, life expectancy of ≥3 months, recovery to National Cancer Institute Common Toxicity Criteria (NCI-CTC; version 4.03) grade ≤1, derived from any prior treatment-related toxicity, and adequate bone marrow, renal, pancreatic, and hepatic functions according to laboratory standard parameters.
