# Supplementary material for: Assessing Prognostic and Predictive Biomarkers of Regorafenib Response in Patients with Advanced Soft Tissue Sarcoma: REGOSARC Study
Source: Cancers (Basel). 2020 Dec 12;12(12):3746. doi: 10.3390/cancers12123746 (PMC7763753; doi:10.3390/cancers12123746)
Supplement: Supplementary file 1 [file cancers-12-03746-s001.pdf]

Assessing Prognostic and Predictive Biomarkers of Regorafenib Response in Patients with Advanced Soft Tissue Sarcoma: REGOSARC Study

Thomas Brodowicz, Bernadette Liegl-Atzwanger, Nicolas Penel, Olivier Mir, Jean-Yves Blay, Karl Kashofer, Axel Le Cesne, Emilie Decoupigny, Jennifer Wallet, Rainer Hamacher, Marie-Cécile Le Deley

Table S1. Frequency of mutation or positivity for the 57 studied biomarkers.

Gene	Assessment technique	Overall N=134	Lipo-sarcoma N=38	Leiomyo-sarcoma N=45	Synovial sarcoma N=17	Others N=34	p
ABL1	HotSpot	0	0	0	0	0	
AKT1	HotSpot	1 (1%)	0	0	1	0	
ALK	HotSpot	0	0	0	0	0	
APC	HotSpot	0	0	0	0	0	
ATM	HotSpot	3 (2%)	0	2	0	1	
BRAF	HotSpot	1(1%)	0	0	0	1	
CDH1	HotSpot	0	0	0	0	0	
CDKN2A	HotSpot	1(1%)	0	0	0	1	
CSF1R	HotSpot	0	0	0	0	0	
CTNNB1	HotSpot	2(1%)	0	0	2	0	
EGFR	HotSpot	0	0	0	0	0	
ERBB2	HotSpot	0	0	0	0	0	
ERBB4	HotSpot	0	0	0	0	0	
EZH2	HotSpot	0	0	0	0	0	
FBXW7	HotSpot	1(1%)	1	0	0	0	
FLT2(1)	HotSpot + FCS(2)	2 (1%)	1	0	0	1	
FGFR2	HotSpot	0	0	0	0	0	
FGFR3	HotSpot	1(1%)	1	0	0	0	
FLT1 (1)	FCS	5 (4%)	2	1	0	2	
FLT3(1)	HotSpot	0	0	0	0	0	
FLT4 (1)	FCS	5 (4%)	2	1	1	1	
GNA11	HotSpot	0	0	0	0	0	
GNAQ	HotSpot	0	0	0	0	0	
GNAS	HotSpot	0	0	0	0	0	
HNF1A	HotSpot	0	0	0	0	0	
HRAS	HotSpot	1 (1%)	0	0	0	1	
IDH1	HotSpot	0	0	0	0	0	
IDH2	HotSpot	0	0	0	0	0	
JAK2	HotSpot	0	0	0	0	0	
JAK3	HotSpot	0	0	0	0	0	
KDR (1)	HotSpot + FCS(3)	7 (5%)	2	2	0	3	
KIT	HotSpot + FCS(4)	1 (1%)	0	0	0	1	
KRAS	HotSpot	0	0	0	0	0	
MET	HotSpot	2 (1%)	0	1	0	1	
MLH1	HotSpot	0	0	0	0	0	
MPL	HotSpot	0	0	0	0	0	
NOTCH1	HotSpot	0	0	0	0	0	
NPM1	HotSpot	0	0	0	0	0	
NRAS	HotSpot	1 (1%)	0	0	0	1	
PDGFRA	HotSpot	0	0	0	0	0	
PDGFRB	FCS	7 (5%)	3	2	0	2	
PIK3CA	HotSpot	3 (2%)	2	0	0	1	

Gene	Assessment technique	Overall N=134	Lipo-sarcoma N=38	Leiomyo-sarcoma N=45	Synovial sarcoma N=17	Others N=34	p
PTEN	HotSpot	2 (1%)	2	0	0	0	
PTPN11	HotSpot	0	0	0	0	0	
RAF1	FCS	2 (1%)	1	0	0	1	
RB1	HotSpot	3 (2%)	1	1	0	1	
RET	HotSpot	0	0	0	0	0	
SMAD4	HotSpot	0	0	0	0	0	
SMARCB1	HotSpot	1 (1%)	0	0	0	1	
SMO	HotSpot	0	0	0	0	0	
SRC	HotSpot	0	0	0	0	0	
STK11	HotSpot	0	0	0	0	0	
***TEK* (*TIE2*)[(1)]**	FCS	3 (2%)	1	1	0	1	
TP53	HotSpot + FCS[(5)]	35 (26%)	5	16	1	13	0.009
***VHL*[(1)]**	HotSpot	0	0	0	0	0	
***VSX2* (*RET1*)**	FCS	2 (1%)	1	0	1	0	
**Angiogenesis pool[(1)]**		20 (15%)	7	5	1	7	0.42
**H19**	RNA in situ hybridization on TMA	24 (18%)	9	5	7	3	0.02

FCS: Full coding sequence analysis; TMA: Tissue Micro-Array; (1) The "Angiogenesis pool" includes *FLT1* (*VEGFR1*), *FLT2* (*FGFR1*), *FLT3*, *FLT4* (*VEGFR3*), *KDR* (*VEGFR2*), *TEK* (*TIE2*), and *VHR*; (2) For *FGFR1* (*FLT2*), studied via both techniques, 2 tumors were found mutated, only via FCS; (3) For *KDR* (*VEGFR2*), studied via both techniques, 7 tumors were found mutated, 2 via both techniques, and 5 only via FCS; (4) For *KIT*, studied via both techniques, 1 tumor was found mutated, via both techniques; (5) For *TP53*, studied by both techniques, 35 tumors were found mutated, 29 via both techniques and 6 only by FCS.

© 2020 by the authors. Licensee MDPI, Basel, Switzerland. This article is an open access article distributed under the terms and conditions of the Creative Commons Attribution (CC BY) license (http://creativecommons.org/licenses/by/4.0/).
